# Donkey’s Milk in the Management of Children with Cow’s Milk protein allergy: nutritional and hygienic aspects

**DOI:** 10.1186/s13052-019-0700-4

**Published:** 2019-08-17

**Authors:** Lucrezia Sarti, Mina Martini, Giovanni Brajon, Simona Barni, Federica Salari, Iolanda Altomonte, Giuseppe Ragona, Francesca Mori, Neri Pucci, Giada Muscas, Fina Belli, Franco Corrias, Elio Novembre

**Affiliations:** 1 Allergy Unit, department of Paediatrics, Anna Meyer Children’s University Hospital, Florence, Italy; 2 Nutrition Unit, department of Paediatrics, Anna Meyer Children’s University Hospital, Florence, Italy; 3 Department of Veterinary Science, University of Pisa, Pisa, Italy; 4 Interdepartmental Research Center Nutraceuticals and Food for Health, University of Pisa, Pisa, Italy; 5 Istituto Zooprofilattico Sperimentale del Lazio e della Toscana M. Aleandri, Florence, Italy

**Keywords:** Children, cow’s milk allergy, donkey milk, Hygienic risk, Nutritional, Nutraceutical

## Abstract

**Background:**

The therapeutic strategy for children with cow’s milk allergy (CMA) consists in the elimination of cow’s milk (CM) from their diet. Donkey’s milk (DM) has been reported to be an adequate alternative, mainly to his nutritional similarities with human milk (HM) and excellent palatability. The aim of present prospective study was to evaluate the nutritional impact of DM on the diet of children with CMA in term of children growth.

**Methods:**

Before the nutritional trial on children and during the study the health and hygiene risks and nutritional and nutraceuticals parameters of DM were monitored. Children with CMA were identified by the execution of in vivo and in vitro tests for CM and subsequent assessment of tolerability of DM with oral food challenge (OFC). Finally, we prescribed DM to a selected group of patients for a period of 6 months during which we monitored the growth of children. A total of 81 children, 70 with IgE mediated cow’s milk protein allergy (IgE-CMPA) and 11 with Food Protein Induced Enterocolitis Syndrome to CM (CM-FPIES), were enrolled.

**Results:**

Seventy-eight out of 81 patients underwent the OFC with DM and only one patient with IgE-CMPA (1.5 %) reacted. Twenty-two out of 81 patients took part of the nutritional trial. All the 22 patients took and tolerated the DM, moreover DM did not change the normal growth rate of infants.

**Conclusions:**

In conclusion, DM resulted safe in term of health and hygiene risks and nutritionally adequate: no negative impact on the normal growth rate of children was assessed. Therefore, it may be a suitable alternative for the management of IgE mediated CMA and FPIES, also in the first 6 months of life, if adequately supplemented.

## Background

Human milk (HM) is the exclusive or primary supply in the first months of a new-born’s life [1], but in cases where it is not available it becomes essential to provide a suitable alternative. Cow’s milk (CM) based formulas are widely used as a substitute for HM, however 2–3% of children present an IgE-mediated cow’s milk protein allergy (IgE- CMPA) [2, 3] and it is also known that in the 0.34% of children, CM can cause the Food Protein Induced Enterocolitis Syndrome (CM-FPIES) [4].

The therapeutic strategy for children with IgE-CMPA or CM-FPIES consists of the total elimination of cow’s milk protein (CMP) from their diet [4, 5, 7]. During the first years of life, milk represents an important source of nutrients, so it’s difficult to eliminate from the everyday diet. Therefore, one of the major objectives of paediatric allergists is to find an appropriate alternative with a pleasant taste, good nutritional values, and hypoallergenic properties that will not induce cross-reactivity with CM [6]. The current guidelines [2, 8–13] recommend extensively hydrolyzed formulas (eHFs) as the first choice with IgE-CMPA treatment except for the more severe reactions where free amino acid formulas (FAAFs) are preferable. Unfortunately, eHFs and FAAFs are hampered by their unpleasant taste not only related to the hydrolysis itself but also to their particular composition (eg fatty acid profile) and by the possibility of residual allergenicity [14–17]. While soy infant formula can be considered a good additional alternative choice because it is readily available, has an acceptable taste, and ensures proper growth in children, it is not recommended as a first choice either for IgE-CMPA treatment, especially in infants younger than 6 months, because of the major risk of developing allergy to soy [18, 19], or for CM-FPIES treatment because a large percentage of these infants can also react to soy [20–22].

Donkey’s milk (DM) has recently received growing interest as has been reported to be an adequate alternative for children with CMPA and CM-FPIES, mainly due to its nutritional similarities with human milk [23] and excellent palatability and tolerability [24–29], unlike the milk of other species, such as goat’s and sheep’s milk, which can lead to cross-reactivity between their proteins and CM proteins [17, 30, 31]. In fact, DM shows a protein fraction more similar to HM than CM, in addition to which, the primary structure of DM’s caseins presents significant differences compared to other species, and it is always more closely related with HM counterparts [18, 32–34]. This may contribute towards explaining the less allergenic properties of DM and its greater digestibility [35]. Furthermore, the high lactose content of DM confers good palatability.

The stimulatory effect of lactose on intestinal calcium absorption - known for its important role in bone mineralization - has been observed in animal models [36], while there are contradictory reports in humans [37].

Among other positive properties, DM also has a high content of lysozyme which, together with immunoglobulins, lactoferrin, and lactoperoxidase, exerts both an immunoregulatory and an anti-tumour activity, and it may also act on the digestive tract by reducing the incidence of gastro-intestinal infections [18, 25, 31, 32, 38].

However, DM has a low-fat content, which corresponds to a low energetic value [25]. While the low lipid content of DM can be considered an advantage in low calorie diets or when a low intake of animal fat is recommended, it may represent a limit in children who require an adequate intake of lipids. In fact, lipids represent 50% up to 12 months and about 40% between 12 months and 24 months of age of daily caloric needs; therefore, if donkey milk is administered as the sole source of nutrition, it must be adequately supplemented with lipids.

The number of studies that focus on the hygiene and health characteristics of DM is increasing [39]. There are reports that show the interactivity of lysozyme and lactoferrin may affect the antimicrobial properties of DM [40] and that the consumption hazards of DM are lower than for CM, especially for microorganisms like enterotoxigenic *E. coli* and thermo-tolerant Campylobacter [41]. Moreover, the low prevalence of mastitis agents in DM has been demonstrated [39, 42]. As pathogenic bacteria and DNA from protozoa have been found in DM [42, 43], due to its use in sensitive consumers, heat treatment of raw milk is recommended to avoid the risk of food-borne diseases. Pasteurisation guarantees both the preservation of the milk’s nutritional properties and the elimination of any pathogenic microorganisms that could be present in raw milk.

The main purpose of this study is to evaluate the nutritional impact of DM, appropriately integrated, on the diet of patients with IgE-CMPA and CM-FPIES in terms of children’s growth. For this purpose a multidisciplinary and prospective study tested the nutritional and nutraceutical characteristics and sanitation of DM, as well as its palatability and tolerability.

## Methods

DM was supplied from a farm located in central Italy, where about 160 Amiata donkeys are reared outdoors, in a semi-intensive system and routinely machine milked twice a day. The farm has been recognised according to European Union (EU) regulation 853/2004.

Before the nutritional trial on children and during the study, the health and hygiene risks and nutritional and nutraceuticals parameters were monitored by the Istituto Zooprofilattico Sperimentale del Lazio e della Toscana (Florence section-Florence, Italy) and the Department of Veterinary Sciences of the University of Pisa (Italy) respectively. The palatability and tolerability of the milk were assessed by the Department of Allergy of the Anna Meyer Children’s Hospital (Florence, Italy): a specific allergological work-up that included skin tests, in vitro tests and oral provocation tests with DM was performed in a day-hospital setting in children with IgE-CMPA or CM-FPIES. The Department of Allergy and Professional dietetic Unit also drew up nutritional plans that included DM, adapted to the needs of patients with IgE-CMPA and CM-FPIES in relation to their age, sex and disease. The same departments monitored the palatability of DM and the growth and the quality of life of the children enrolled in the study for a period of 6 months.

### Evaluation of the health hazards of DM consumption and nutritional and nutraceutical analyses

The health and hygiene risk analyses were carried out on 36 bulk milk samples (18 of raw milk and 18 of the corresponding milk pasteurised at 65 °C for 30 min) taken monthly, while the nutritional analysis regarded the pasteurised samples. All the samples were taken to the laboratories in tanks at 4 °C; no preservatives were added. Microbiological analyses required by European (EC Regulation 853/2004) and Italian national legislation (Intesa Stato-Regioni 25 January 2007) were conducted on the raw milk samples. In particular, the hygienic quality of the milk was studied by determining the Total Viable Count (TVC) at 30 °C [UNI EN ISO (Italian National Unification Body), 4833–1: 2013], and the food safety via the occurrence of the main pathogenic bacteria responsible for food-borne infections: Salmonella spp. (ISO 6579:2002/Cor 1: 2004), *Listeria monocytogenes* (UNI EN ISO 11290-1: 2005), Campylobacter spp. (UNI EN ISO 11290-1: 2005) and coagulase-positive Staphylococci (ISO 6888-2: 1999/Amd 1:2003). Furthermore, TVC and Enterobacteria (ISO 21528-2: 2004a) (process hygiene criterion provided for by EC Regulation 2073/2005), and coagulase-positive Staphylococci were performed on the pasteurised milk samples. The occurrence of *L. monocytogenes* was also determined in the pasteurised milk samples, as required by the HACCP manual of the farm.

All the samples were analysed for dry matter and lactose content via infrared analysis (Milkoscan, Italian Foss Electric, Padova, Italy); proteins, caseins and ashes [44]. Fat was gravimetrically determined after extraction as per the Rose-Gottlieb method [45]. The individual mineral content (Ca, P, Mg, K, Na, Zn) (mg/L) was determined by atomic absorption spectroscopy and ultraviolet–visible spectroscopy according to the AOAC (2000) [46], and Murthy and Rhea (1967) [47]. Methyl esters of fatty acids for gas chromatographic analysis were prepared using methanolic sodium methoxide according to Christie (1982) [48]. The gas chromatographic analysis of the milk was conducted as described in the paper by Ragona et al., 2016 [39].

For the Vitamin D quantification, lipids from 75 ml of DM were saponified by adding KOH pellets directly to the milk according to Perales et al. (2005) [49] at 40 °C for 32 min. Ethanol and double distilled water were then added to the sample in order to remove the polar compounds and prevent foaming. Afterwards the solution was transferred into a 500-mL separatory funnel, and an initial extraction of the unsaponifiable fraction was performed using 75 ml hexane. The aqueous phase was thus drained and collected in order to repeat two extractions by adding 75 ml of hexane each time and the organic phase from both was collected in a rotavapor flask. Finally, the organic phase was evaporated to dryness on a rotary evaporator and the extract re-suspended in 500 μl of acetonitrile and filtered through a 0.45-μm diameter syringe filter. 100 μl of the extract were injected into an HPLC and isocratically eluted using acetonitrile-methanol 97: 3 as a mobile phase at a flow of 1 ml/min, as described by Hagar et al. (1994) [50]. A Kinetex core-shell column (Phenomenex, Inc. A) was used as the stationary phase and the UV detector was set at 254 nm. Cholecalciferol and ergocalciferol in the milk samples were quantified by comparison with a calibration curve obtained via the injection of the pure standards (Sigma Chemical Co., St. Louis).

The activity of the lysozyme was assessed by the Fluorimetric method on a microplate (EnzChek Lysozyme Kit, Invitrogen, Carlsbad CA, USA), measured by means of a Spectrofluori Meter (Ascent, Thermo Labsystem FL, USA) and expressed in units/ml.

### Allergological work-up and palatability assessment

This was a prospective study that recruited 81 children referred to the Allergy Unit of the Anna Meyer Children’s Hospital: 70 children (45 males, 25 females; age-range 6 months − 18 years, mean 5.2 ± 5.3 years) with proven IgE-CMPA and 11 patients with proven IgE-FPIES (4 males, 7 females; age-range 3 months – 8 months, mean 4.73 ± 1.68 months).

The allergological work-up included: skin prick test (SPT), specific serum IgE (s-IgE) and oral food challenge (OFC) with DM. The OFC was performed in patients with IgE CMPA according to the DRACMA guidelines and to the protocol of Leonard et al. in patients with IgE-FPIES [29, 51–53].

While performing the OFC we also evaluated the palatability of the DM: in children ≥3 years of age, DM palatability was assessed with a specific Wong-Baker modified pain scale while in children < 3 years of age it was assessed through the physician’s judgment [53]. Before beginning the study informed parental consent was given.

### Development of nutritional plans and monitoring growth

The Department of Allergy and the Professional dietetic Unit of the Anna Meyer Children’s Hospital drew up nutritional plans which included DM and were appropriate for the needs of 16 out of the 70 patients with IgE-CMPA (12 M: 4 F) and 6 out of the 11 patients with CM-FPIES (4 M: 2 F), who referred to the Allergy Unit.

On the basis of the DM nutritional analyses, a number of different nutritional plans were formulated with an appropriate daily calorie intake depending on the age of the patients (Table 1). For children older than 3 years in which milk accounts for less than 10% of calories, we estimated that its replacement with DM does not give rise to significant variations. The daily-prescribed dose of DM varied depending on age (Table 1). DM was provided free of charge by the Meyer Children Hospital to all children enrolled for the six-month study period. In addition, we prescribed vitamin D (cholecalciferol, Vitamin D3) in specific doses for each age and included a supplement of fat content in the nutritional plans due to the low-fat content in DM (Table 1). Lipid supplementation consisted of 3 g of lipids for every 100 ml of donkey milk taken, in the form of extra virgin olive oil for children over 6 months of age, to be added either to the milk itself or to savoury meals; in infants younger than 6 months the aforementioned lipid supplementation is half represented by extra virgin olive oil and half by a gluco-lipid supplement to be mixed with donkey’s milk. The gluco-lipidic supplement contains 40% of MCT lipids in its lipid fraction. Also, Vitamin D supplement was provided free of charge during the period of the study.

**Table 1:** The nutritional plans formulated for the 22 patients enrolled in the nutritional trial

Age of patients	Number of nutritional plans	Prescribed daily dose of donkey milk	Vitamin D supplement	Fat addition to the diet
< 6 mo^a^	5^b^	500–1000 ml according to the age (from 3 months to one year).	2600 UI/week	3 g of fat in each 100 ml of DM [1.5 g of Extra Virgin olive Oil (EVO) and 1.5 g of Medium Chain Triglycerides vegetable oil (MCT oil)]
6–12 mo	1			Addition of fat to the daily meals (for example EVO at lunch and dinner or a snack in the afternoon to be eaten together with bread).
1–3 y	1	200–250 ml	4000 UI/week.	
3–6 y	1			

*DM* donkey milk, *mo* months, *y* years

^a^un-weaned infants; ^b^ one for each month of age

The patients enrolled in the study met with a dietician three times during the six-month study period: at the beginning (T0) and at the end of the study (T1) in order to monitor the nutritional conditions. The patients were also evaluated at 3 months to monitor the compliance of the nutritional plan with its supplements. During the first visit, the dietician explained to parents the nutritional plan specifically designed for their children, the nutritional, nutraceutical and hygienic characteristics of Amiatina DM, the importance of the supplements of fat content and vitamin D, the methods of DM supply and procedures for storing and consuming DM at home. The dietician together with the paediatric allergist also explained the correct reading of the labels to avoid accidentally taking of CM. During the follow-up period the parents kept a food diary, recording daily DM consumption and the related fat and vitamin D supplements, in order to implement changes to the nutritional plan, where necessary.

In addition, the nutritional status of the patients was assessed with blood biochemical parameters and auxological parameters. The biochemical parameters of nutritional interest measured included: blood count (in particular red blood cells and haemoglobin), serum albumin, 25-OH vitamin D level, azotaemia and thyroid function tests (TSH, fT4). Blood was drawn in the Allergy Unit at the beginning of the study (T0) and after 6 months of DM consumption (T1). The nutritional state was also evaluated with auxological parameters which considered weight and body length for children and infants up to 2 years, and stature thereafter. Weight was measured with electronic integrating scales (SECA 757; Hamburg, Germany: precision ±1.0 g). Supine length was measured with a Harpenden infant meter and stature was measured with a Harpenden stadiometer. The weight was expressed in kilograms (kg) and the growth curves used were those of the World Health Organisation (WHO) for children and infants up to 2 years of age [54], and the Central Disease Control (CDC) for children over 2 years of age [55]. The length/height was expressed in centimetres (cm) and the growth curves used were those of the WHO for children up to 2 years of age and those of the CDC for children over 2 years of age. The auxological parameters were collected at T0 and T1.

Moreover, weight and length/stature-gain were evaluated in terms of Z-score This method evaluates changes in anthropometric parameters associated with the introduction of DM. Z-score for weight and length/stature-gain were calculated at T0 and T1 in the 16 patients with IgE-CMPA and the 6 patients with CM-FPIES. We also focused on the weight and length/stature- gain in terms of Z-score at T0 and T1 in the patients younger than 1 year in which milk consumption is relevant.

### Statistical analysis

Health data and chemical composition of DM are reported as mean and standard deviation (SD). Z-scores of weight and length/stature for age were calculated from the formula Z = x-|X|/|SD|, taking the Gardner and Pearson growth curves as reference for children up to 2 years and the Tanner curves after 2 years of age. Z-score values obtained between check-ups were analysed with the paired t-test. Significance was established with the paired t-test, with *p* < 0.05 as cut-off. The data were analysed using a commercially available statistical software package (SPSS, Chicago, IL, USA).

## Results

### Evaluation of the health hazards of DM consumption and nutritional and nutraceutical analyses

In raw and pasteurised milk samples, the TVC was on average 74,333.33 CFU/mL (±34,416.57) and 4332.22 CFU/mL (±3046.78) respectively. In raw milk samples, the bacteria responsible of food-borne outbreaks (Salmonella spp., *Listeria monocytogenes*, Campylobacter spp.) were never detected. Moreover, coagulase-positive Staphylococci were found in the raw milk with a count ranging from < 1 CFU/mL to 190 CFU/mL, and an average value of 133.83 CFU/mL. The Enterobacteria count was always lower than 1 CFU/mL in the pasteurised milk samples in compliance with Regulation (EC) No 1441/2007 and *L. monocytogenes* was never isolated.

DM showed a dry matter percentage of 9.32, of which the major component was lactose with a percentage of 7.05, whereas 0.81 was the percentage of casein; fat and ash percentages were 0.31 and 0.37 respectively (Table 2). Among the minerals, calcium and potassium were present in higher quantities (633.31 and 653.32 mg/L respectively) while zinc was 3.16 mg/L.

**Table 2:** Chemical composition of pasteurized donkey’s milk

Item	Units	Mean	Standard Deviation
Fat	%	0.31	0.053
Protein	%	1.59	0.137
Casein	%	0.81	0.134
Dry Matter	%	9.32	0.285
Ash	%	0.37	0.022
Lactose	%	7.05	0.150
Ca	mg/L	633.31	137.440
P	mg/L	386.31	69.21
K	mg/L	652.32	73.329
Mg	mg/L	92.59	27.737
Na	mg/L	168.20	72.420
Zn	mg/L	3.16	1.500
Total Vit. D	ug/100 ml	1.97	0.454
Vit. D2	ug/100 ml	1.72	0.833
Vit. D3	ug/100 ml	0.25	0.184
Lysozyme activity	U/ml	1402	286.658

As regards the milk fatty acid profile, the most frequently represented fatty acids were palmitic acid (22.10 g/100 g of fat), oleic (21.58 g/100 g of fat), and linoleic (11.18 g/100 g of fat) (Table 3). Saturated and unsaturated fatty acids were 52 and 48 g/100 g of fat respectively. Among the unsaturated, n-3 fatty acids were about 8 g/100 g of fat and the major n-3 component present in milk was linolenic acid (7.52 g/100 g of fat), whereas linoleic was the main n-6 fatty acid; n-3/n-6 ratio was 0.72. DM showed a mean lysozyme activity of 1402 + 286.658 U/ml and total content of vitamin D1.97 μg/100 ml principally due at vitamin D2 (Table 2).

**Table 3:** Fatty acid composition of pasteurized donkey’s milk (g/100 g of fat)

Item	Mean	Standard deviation	Item	Mean	Standard deviation
C4:0	0.08	0.021	C18:2 t-9.12	0.08	0.066
C6:0	0.23	0.104	C18:2 c-9.12	11.18	1.904
C8:0	3.56	0.762	C18:3 n6	0.13	0.078
C10:0	8.11	1.403	C20:0	0.17	0.098
C11:0	1.31	0.316	CLA c9. t11	0.07	0.045
C12:0	7.66	1.145	C20:1	0.13	0.090
C13:0	0.05	0.031	C21:0	0.08	0.096
C14:0	6.33	1.061	C20:2	0.13	0.066
C14:1	0.40	0.114	C20:3n3	0.21	0.060
C15:0	0.39	0.075	C20:3 n6	0.07	0.061
C15:1	0.17	0.098	C22:0	0.06	0.042
C16:0	22.10	2.924	C22:1	0.25	0.052
C16:1	3.86	0.840	C20:4n6	0.07	0.035
C17:0	0.26	0.100	C23:0	0.04	0.038
C17:1	0.40	0.098	C22:2	0.10	0.087
C18:0	1.66	0.357	C20:5	0.07	0.060
C18:1 *t-9*	0.04	0.004	C24:0	0.09	0.067
C18:1 *t-11*	0.05	0.035	C24:1	0.06	0.070
C18:1 c-9	21.58	2.904	C22:5	0.10	0.084
C18:1 c-11	1.08	0.273	C22:6	0.05	0.041
C18:3n3	7.52	2.494			
SCFA (≤C10)	11.97	2.131	SFA	52.17	3.987
MCFA(≥C11 ≤ C17)	42.93	4.155	MUFA	28.05	3.487
LCFA(≥C18)	45.10	3.731	PUFA	19.79	2.433
n3/n6 ratio	0.72	0.274	UFA/SFA	0.93	0.141

*SCFA* short-chain fatty acids, *MCFA* medium-chain fatty acids, *LCFA* long-chain fatty acids, *SFA* saturated fatty acids, *MUFA* monounsaturated fatty acids, *PUFA* polyunsaturated fatty acids, *UFA* unsaturated fatty acids

### Allergological work-up and palatability assessment

The DM was well tolerated and showed good palatability both in patients with IgE-CMPA and CM-FPIES. In particular, 67 out of 70 patients with IgE-CMPA underwent the OFC, the parents of the others 3 patients refused to give their consent because of positivity to the SPT and/or s-IgE to DM. Only one out of 67 (1.5%) patients with IgE-CMPA reacted to the DM. Of the patients with CM-FPIES, 11 out of 11 (100%) underwent the OFC and all patients tolerated the DM. In general, 77 out of 78 patients (98.7%) that underwent OFC with DM tolerated it.

### Nutritional plans, monitoring growth and quality of life

Sixteen out of 66 patients with IgE-CMPA and six out of 11 patients with CM-FPIES took and tolerated the DM for the prescribed length of time. All 22 patients also followed the nutritional plans formulated for each one, without significant variations in the quantity of DM consumed.

The mean age (± SD) of the patients with IgE-CMPA at T0 was 20 (± 18.4) months (range 9–79 months). The mean weight (±SD) was 9.993 (± 4.660) kg and the mean length/stature (±SD) was 77.41 (± 17.59) cm at T0. The mean weight (±SD) was 12.160 (± 3.087) kg and the mean length/stature (±SD) was 87.91 (± 25.77) cm at T1. As regards the six patients with CM-FPIES, the mean age (±SD) at T0 was 5.33 (± 1.75) months (range 3–8 months), the mean weight (±SD) was 7.566 (± 3.130) kg and the mean length/stature (±SD) was 66 (± 3.93) cm. The mean values (±SD) for weight and length/stature at T1 were 9.470 (± 5.194) kg and 74.4 (± 38.5) cm respectively.

Tables 4 and 5 report the variations in the auxological values (expressed with Δ z-score) in the 22 patients enrolled at T0 and T1 grouped for pathology (Table 4) and in the children younger than 1 year (Table 5).

**Table 4:** Variation of z score for weight and length/stature during DM assumption

IgE- CMPA	T0	T1
Number of patients	16	16
Δz-score for the weight	−0.64	−0.03 (+ 0.61)
Δz-score for the length/stature	−0.80	0.12 (+ 0.92)*
CM-FPIES	T0	T1
Number of patients	6	6
Δz-score for the weight	0.16	0.25 (+ 0.09)
Δz-score for the length/stature	0.11	0.12 (+ 0.01)

*P < 0.05

DM donkey milk; IgE- CMPA: IgE-mediate cow’s milk protein allergy; CM-FPIES: Food Protein Induced Enterocolitis Syndrome T0: mean values of z-score at the beginning of the donkey milk assumption; T1: mean values of z-score after 6 months of the donkey milk assumption; Δz-score: variation of the mean values of z score

**Table 5:** Variation of z score for weight and length/stature in the patient younger than one year

0–12 months	T0	T1
Number of patients	17	17
Δz-score for the weight	−0.31	+ 0.25 (+ 0.56)
Δz-score for the length/stature	−0.62	+ 0.25 (+ 0.87) *

*P < 0.05

T0: mean values of z-score at the beginning of the donkey milk assumption; T1: mean values of z-score after 6 months of the the donkey milk assumption; Δz-score: variation of the mean values of z score

At T0, patients with IgE-CMPA had negative weight and length/stature Z-scores and showed an improvement at T1, statistically significant for length/stature Z-score (*p* < 0.05). Similarly, we found a statistically significant improvement for length/stature Z-score in patients younger than 1 year (Table 5). The growth in weight was similar to that of the reference population both in IgE-CMPA and in infant younger than 1 year. The infants with CM-FPIES showed a normal nutritional status from the beginning of enrolment and maintained it during the 6 months of being fed DM, with a good increase in weight and length/stature similar to the reference population.

The blood biochemical parameters with nutritional interest were evaluated in 19 patients (16 with IgE-CMPA and 3 with FPIES) out of 22 (86.4%), the other 3 patients with CM-FPIES did not perform the blood tests due to refusal by their parents. No relevant variations were observed; in fact, all the blood values were always in the normal range (data not shown).

## Discussion

The TVC of the raw milk was on average lower than the limit required by the Regulation (EC) 853/2004 (≤1.500 × 103 CFU/mL). In addition, TVC was lower than that described in other studies on pasteurised donkey’s milk [52]. Coagulase-positive Staphylococci showed low average values below the limit of 105 CFU/mL which is considered a risk for food safety.

Although *L. monocytogenes* is killed by pasteurisation, it may represent a high food safety hazard in milk not properly pasteurised or contaminated after thermal treatment, especially in vulnerable subjects such as infants and pregnant women. Therefore, a careful risk assessment of *L. monocytogenes* can help ensure the food safety of pasteurised DM. The Enterobacteria count in the pasteurised milk samples was in compliance with Regulation (EC) No 1441/2007.

Compared to milk from other dairy species, DM is the most similar to HM [23]. In particular, the nutritional similarities concern the low content of caseins and ashes, thus limiting allergy and favouring a lower contribution of renal solutes and a high content of lactose that promotes good palatability. Donkey and human milk share a similar unsaturated:saturated ratio [56]. Furthermore, the fat content in donkey’s milk is lower compared other milks, and this implies a low energetic value of the milk [25]. A multidisciplinary group approach, including a dietician, is fundamental in the planning of a “personalized nutritional plan”, to fully satisfy the nutritional needs of patients based on age, symptoms but also food preferences and nutritional behavior of the patient [57] However, our study shows that this limit can easily be overcome with appropriate supplementations.

As already described in two of our previous papers, DM is well tolerated and appreciated by children with CMPA and CM-FPIES [29, 53]. It has long been known that despite similar energy intakes, children with CM allergy have a shorter stature than controls without CM allergy, [58, 59]. Our results are in line with previous studies that show a check-up growth after the introduction of DM in children with CM allergy [25–27]. No relevant variations in terms of blood and metabolic parameters were detected.

In particular, despite major concerns regarding the use of un-modified DM as sole nutritional source (if not adequately supplemented), our results indicate that it could be considered a valid alternative in weaned infants (older than 5–6 months), and also in infants aged between 0 and 6 months with appropriate nutritional supplements. In fact, our study found that DM allowed a regular increase in weight in children aged 0–12 months and an improvement in their length/stature growth.

A very positive aspect deriving from our study was the improvement in the quality of life of the patients and their families. The parents of the children referred to the dietitian that their children/infants were less restless and more relaxed; they ate with more pleasure and showed greater curiosity towards the various foods that were offered. Probably this improvement is mainly due to the exclusion of CM from the diet, but also to the good taste and nutritional characteristics of DM, as well as the dietetic follow-up that we offered to patients and their families. As a result, there was more serenity in the family, less anxiety, and in general, a better quality of life.

A limit in the consumption of DM as a substitute for CM remains its high cost, however, the eHFs and FAAFs are also expensive and, unlike DM, they have low palatability. Our encouraging results should potentiate the production of DM, which could lead to a reduction in its cost.

Moreover despite the encouraging results, limitations of our study were the small number of the enrolled patients and the lack of a control group. More extensive studies are needed regarding the use of DM as a substitute of CM in patients with IgE-CMPA and CM-FPIES and it would be interesting a head-to-head comparison between DM and other nutritional sources in terms of nutritional and allergenic aspects.

## Conclusions

In conclusion DM resulted safe in term of health and hygiene risk and nutritionally adequate: no negative impact on the growth rate of infants and children was assessed. Therefore, it may be a suitable alternative for the management of IgE- CMPA and CM-FPIES, also in the first 6 months of life, if adequately supplemented.

## Data Availability

The data that support the findings of this study are available from the authors.

## Abbreviations

CDC: Central Disease Control
CM: Cow’s milk
CM-FPIES: Food Protein Induced Enterocolitis Syndrome
CMP: cow’s milk protein
DM: donkey milk
eHFs: extensively hydrolyzed formulas
FAAFs: free amino acid formulas
HM: Human milk
IgE- CMPA: IgE-mediate cow’s milk protein allergy
OFC: oral food challenge
s-IgE: specific serum IgE
SPT: skin prick test
WHO: World Health Organisation
